# Serum anti-SPP1 autoantibody as a potential novel biomarker in detection of esophageal squamous cell carcinoma

**DOI:** 10.1186/s12885-022-10012-9

**Published:** 2022-08-29

**Authors:** Chen Wang, Guiying Sun, Huimin Wang, Liping Dai, Jianying Zhang, Renle Du

**Affiliations:** 1 School of Basic Medical Sciences & Henan Institute of Medical and Pharmaceutical Sciences, Academy of Medical Science, Zhengzhou University, Zhengzhou, 450052 Henan China; 2 State Key Laboratory of Esophageal Cancer Prevention & Treatment, Zhengzhou University, Zhengzhou, 450052 Henan China; 3 Henan Key Laboratory of Tumor Epidemiology, Zhengzhou University, Zhengzhou, 450052 Henan China; 4 College of Public Health, Zhengzhou University, Zhengzhou, 450052 Henan China; 5 Henan Key Medical Laboratory of Tumor Molecular Biomarkers, Zhengzhou University, Zhengzhou, 450052 Henan China

**Keywords:** SPP1, Tumor-associated antigen, Autoantibody, Detection, Esophageal squamous cell carcinoma

## Abstract

**Background:**

Esophageal squamous cell carcinoma (ESCC) has poor prognosis mainly due to lacking of effective diagnostic biomarkers. Aberrant expression of secreted phosphoprotein 1 (SPP1) protein has been observed in several cancers. The purpose of this study is to assess the feasibility of serum autoantibody to SPP1 in detection of ESCC.

**Methods:**

The SPP1 protein levels in 108 ESCC tissues and 72 adjacent normal tissues were analyzed by immunohistochemistry. Discovery group containing 62 serum samples from ESCC patients and 62 serum samples from normal controls (NC) were used to detect the levels of anti-SPP1 autoantibody by enzyme-linked immunosorbent assay (ELISA). Validation group containing another 100 ESCC and 100 NC serum samples were tested to confirm the levels of autoantibody to SPP1. Western blotting was performed to further confirm the results of ELISA.

**Results:**

SPP1 protein was significantly overexpressed in ESCC tissues compared to adjacent normal tissues. ELISA results showed that serum autoantibody to SPP1 was significantly increased in ESCC compared to NC in both discovery and validation groups. Autoantibody to SPP1 could discriminate patients with ESCC from NC with the area under curve (AUC) values of 0.653 and 0.739 in discovery and validation group, respectively. The results of ELISA and the occurrence of immunoreactivity to SPP1 in ESCC sera were confirmed by western blotting.

**Conclusion:**

Our study indicated the potential significance of anti-SPP1 autoantibody as a novel biomarker for detection of ESCC.

**Supplementary Information:**

The online version contains supplementary material available at 10.1186/s12885-022-10012-9.

## Introduction

Esophageal cancer (EC) is a common cancer and threatens the health of people, which ranks the seventh in terms of incidence (604,000 new cases) and the sixth in the leading mortality (544,000 deaths) around the world in 2020 [1]. In China, there were 477,900 new patients and 375,000 deaths of EC which ranked the third cause of cancer-related death [2]. EC can be classified into two major histologic types containing ESCC and esophageal adenocarcinoma (EAC). More than 80% of global ESCC patients was diagnosed in Asia [3].

Despite many advances in the diagnosis and treatment of ESCC, patients with ESCC are usually diagnosed at an advanced stage (III and IV), who have a 5-year survival rate less of 15% and the prognosis is quite poor [4, 5]. However, the 5-year survival rate could reach more than 80% when patients with ESCC are diagnosed at an early stage (I and II) and receive timely treatment [6]. The traditional methods of diagnosing ESCC include mucosa biopsy and endoscopy examination, but these methods are expensive and invasive [7]. Therefore, identifying novel non-invasive biomarkers to improve the diagnosis in ESCC is urgently needed. Previous studies have illustrated that tumor-associated antigens (TAAs) are a category of proteins that aberrantly expressed in cancer, which could elicit the production of autoantibodies to these antigens [8, 9]. Autoantibodies to TAAs in sera from patients are more stable and persistent than other potential biomarkers including the TAAs themselves and can be used as biomarkers in detection of solid tumors [10]. Furthermore, anti-TAAs autoantibodies could be detected at an early stage before the development of clinical symptoms [11]. Therefore, it is crucial to develop anti-TAAs autoantibodies as biomarkers to supplement current screening modalities in detection of ESCC.

SPP1, also known as osteopontin, encoded by the human gene *SPP1* is a cytokine upregulating expression of IFN-γ and IL-12, which is a critical mediator in tumor-associated inflammation and promotes metastasis of cancers [12, 13]. Increasing evidence shows that SPP1 is overexpressed and involved in the progression and poor survival of many types of cancers, including hepatocellular carcinoma [14], glioblastoma [15], breast cancer [16], melanoma [17], colorectal cancer [18]. The overexpression of SPP1 could promote programmed death ligand 1 (PD-L1) expression in HCC, which drives HCC metastasis [14]. High PD-L1 expression is associated with tumor aggressiveness and poor prognosis [19]. In ESCC, down-regulated expression of SPP1 can repress cell motility, cell invasion *in vitro* and tumor formation, lymph node metastasis in nude mice [20]. The five-year survival rate is better in patients without SPP1 expression than that in those with positive SPP1 expression in ESCC. More recently, integrated bioinformatics analysis indicates that the high expression of SPP1 is associated with poor prognosis in ESCC patients [21–23].

It has been reported that anti-SPP1 autoantibody is detected in sera of breast and pancreas cancer [24]. However, to date there is no study investigating whether SPP1 protein induces an autoimmune response in ESCC. Here, we aimed to evaluate the potential significance of serum anti-SPP1 autoantibody as a novel biomarker for ESCC detection.

## Material and methods

### Immunohistochemical (IHC) assay

The ESCC tissue microarrays performed for analyzing the expression of SPP1 protein were obtained from Shanghai Outdo Biotech Co. LTD (No. HEsoS180Su11, Shanghai, China). Additional file 1 showed the clinical information of ESCC patients. Mouse monoclonal anti-SPP1 antibody (1:500 dilution, Abcam, ab69498, Cambridge, UK) was provided as the primary antibody in IHC analysis. Biotin-labeled secondary antibody, the detecting reagents and the specific operations were offered by this company. All the results of IHC analysis were obtained from two independent pathologists. The degree of immunostaining was divided into different scores based on the staining intensity and the percentage of positively stained cells. The staining intensity was graded according to the following criteria: no staining, score 0; weak expression, score 1; moderate staining, score 2; strong staining, score 3. The percentage of staining positive cells was scored as follows: score 0 (0% stained), score 1 (1–25% stained), score 2 (26–50% stained) and score 3 (51–100% stained). The IHC scores were obtained from multiplying the staining intensity scores by the percentage of positive cells scores and ranged from 0 to 9. IHC scores lower than 6 were identified as low expression of SPP1, and scores of 6 to 9 were identified as high expression of SPP1.

### Collection of serum samples

Serum samples of two independent groups were used and detailed characteristics were shown in Table 1. In the discovery group (*n* = 124), there were 62 ESCC serum samples collected from a third-level grade-A hospital (Zhengzhou, China), which were matched 62 normal control serum samples selected from the biological specimen bank of Henan Key Laboratory of Tumor Epidemiology. A separate larger numbers of serum samples from the validation group were used to confirm the results of the discovery group, which consisted of 100 ESCC serum samples and 100 normal controls. All serum samples of ESCC patients who had not received treatments and other malignancies were obtained from new diagnosis by histopathology. All normal controls didn’t have autoimmune and digestive tract-related diseases. The ESCC and NC were matched by 1:1 ratio according to the sex and age (± 5 years). The utilization of human samples was approved by the Ethics Committee of Henan Institute of Medical and Pharmaceutical Sciences, Zhengzhou University. All participants gave written informed consent to participate in this study.

**Table 1:** Characterizations of ESCC patients and normal controls used in study

	Discovery group (*n* = 162)		Validation group (*n* = 200)	
	ESCC	NC	ESCC	NC
N	62	62	100	100
Age				
Mean ± SD	63.5 ± 8.3	62.5 ± 8.1	63.5 ± 8.1	62.3 ± 7.7
Range	44–88	50–83	42–84	43–83
Sex				
Male (%)	43(69.35)	43(69.35)	66(66.00)	66(66.00)
Female (%)	19(30.65)	19(30.65)	34(34.00)	34(34.00)
Smoking				
Yes	22(35.48)		32(32.00)	
None	37(59.68)		62(62.00)	
Unknow	3(4.84)		6(6.00)	
Drinking				
Yes	17(27.42)		28(28.00)	
None	42(67.74)		64(64.00)	
Unknow	3(4.84)		8(8.00)	
Clinical stage				
I	10(16.13)		17(17.00)	
II	10(16.13)		12(12.00)	
III	9(14.52)		23(23.00)	
IV	8(12.90)		8(8.00)	
Unknow	25(40.32)		40(40.00)	
Differentiation				
High	7(11.29)		5(5.00)	
Medium	9(14.52)		19(19.00)	
Low	9(14.52)		21(21.00)	
Unknow	37(61.29)		55(55.00)	
Tumor site				
Upper thorax	14(22.58)		9(9.00)	
Middle thorax	19(30.65)		41(41.00)	
Lower thorax	15(24.19)		21(21.00)	
Unknow	24(38.71)		29(29.00)	
Family tumor history				
Yes	3(4.84)		10(10.00)	
None	55(88.71)		83(83.00)	
Unknow	4(6.45)		7(7.00)	
Lymphatic metastasis				
Positive	17(27.42)		28(28.00)	
Negative	24(38.71)		36(36.00)	
Unknow	21(33.87)		36(36.00)	
Distant metastasis				
Positive	8(12.90)		6(6.00)	
Negative	30(48.39)		55(55.00)	
Unknow	24(38.71)		39(39.00)	

*ESCC* Esophageal squamous cell carcinoma, *NC* Normal controls

### Enzyme-linked immunosorbent assay (ELISA)

The recombinant SPP1 protein was obtained from Cloud-Clone Corp (No. RPA899Hu02, Wuhan, China). The autoantibody to SPP1 protein was detected by ELISA written in detail in our previous study [25]. Briefly, the recombinant SPP1 protein was coated to 96-well microliter plates as antigens at concentration of 0.5 µg/ml. The serum samples were used as the primary antibody at the dilution of 1:100. The secondary antibody was mouse anti-human IgG conjugated horseradish peroxidase (HRP) (Wuhan Aoko Biotechnology Co.LTD), which was diluted at 1:5000. Each plate set six duplicate serum samples as quality control and two blank controls to enable the stability and accuracy of optical density (OD) values in all the plates. The OD values read at 450 nm subtracting from that at 620 nm were used for further analysis.

### Western blotting

The serum samples of ESCC that were positive response to SPP1 in ELISA were detected by western blotting to confirm the occurrence of immunoreactivity in the sera. Mouse monoclonal anti-SPP1 antibody (1:100 dilution, Abcam, ab69498, USA) was regarded as a positive control. The procedure of western blotting utilized in this study was described in our previous study [26]. In brief, the recombinant SPP1 protein was electrophoresed by 10% SDS-PAGE and transferred onto a nitrocellulose membrane that was then cut into strips and incubated with selected sera diluted at 1:100, subsequently incubated with mouse anti-human IgG conjugated HRP diluted at 1:5000.

### Statistical analysis

IBM SPSS Statistics 21.0 and GraphPad 8.0 was used to carry out all statistical analysis. IHC scores of ESCC and adjacent normal tissues were analyzed by independent t test and the correlation between SPP1 expression and clinicopathological features in ESCC patients was performed by χ2 test. Mann–Whitney U test was used to compare differences of the levels of serum autoantibody to SPP1 between ESCC and NC. Differences in positive frequencies of autoantibody to SPP1 between ESCC and NC and in different clinical subgroups were evaluated by the χ2 test. The AUCs of serum autoantibody against SPP1 protein was used for distinguishing ESCC from NC by receiver operating characteristic (ROC) analysis. De Long test was used to analyze AUCs of different clinical subgroups. The relationship between clinicopathological factors and anti-SPP1 autoantibody in patients with ESCC was evaluated by the analysis of independent t test. Mean plus standard deviation (SD) of OD values from NC was regarded as cut-off value. As *P* value was less than 0.05, the test results were considered statistically significant. All *P* values were calculated based on two sides.

## Results

### SPP1 protein was highly expressed in ESCC tissues

**Fig. 1:**
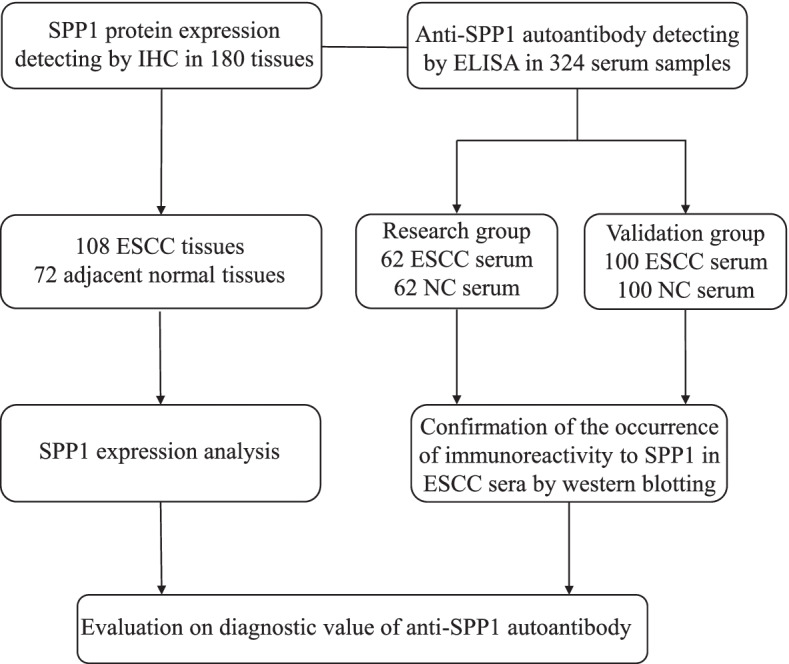
The overall study design. ESCC, esophageal squamous cell carcinoma; NC, normal control; ELISA, enzyme-linked immunosorbent assay; IHC, immunohistochemistry

**Fig. 2:**
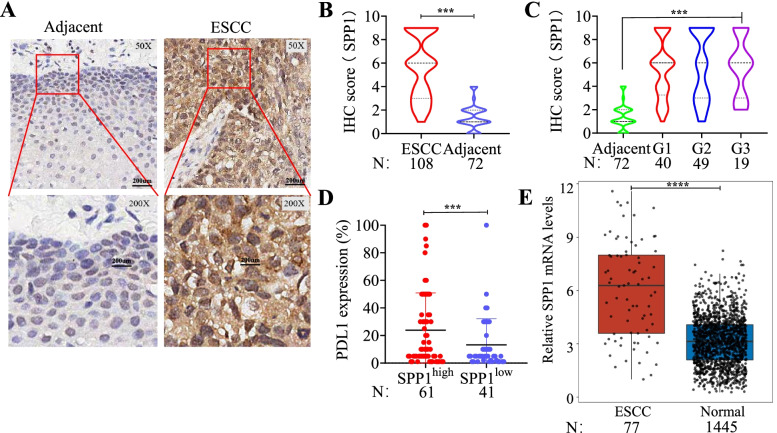
SPP1 was highly expressed in human ESCC tissues. **A** Representative IHC staining images of SPP1 in adjacent normal tissue and tumor tissue from ESCC tissue microarray. **B** Statistical analyses of the IHC scores of SPP1 expression in ESCC tissues and adjacent normal tissues. **C** The expression profiles of SPP1 in different pathological grades of ESCC tissues. **D** The expression levels of PDL1 in SPP1^high^ and SPP1^low^ groups. **E** Transcript levels of SPP1 in 1445 normal tissues and 77 ESCC primary tumors from TCGA and GTEx databases. ESCC, esophageal squamous cell carcinoma; TCGA, The Cancer Genome Atlas; GTEx, Genotype-Tissue Expression. ***P < 0.01; ****P < 0.001

The overall design of this study was presented in Fig. 1. The SPP1 protein levels in 108 ESCC tissues and 72 adjacent normal tissues were analyzed by IHC. SPP1 protein was strong positive staining in a representative ESCC tissue compared with weak staining in a paired adjacent normal esophageal tissue (Fig. 2A). According to the final scores of IHC, SPP1 protein was significantly higher in ESCC tissues than that in paired adjacent tissues (*n* = 72) (Fig. 2B). Based on pathological grades (G1, G2 and G3), SPP1 protein was significantly overexpressed in ESCC tissues compared to adjacent normal tissues (Fig. 2C). Higher PDL1 expression level was observed in ESCC patients with higher SPP1 expression (Fig. 2D). Microscopy images of immunohistochemistry from ESCC tissue microarray ESCC are presented in Supplementary Fig. 1. Table 2 showed the correlation between SPP1 expression and clinicopathological features in ESCC patients, including sex, age, tumor size, lymphatic metastasis and clinical stage, which showed no significant difference. We next explored the mRNA levels of SPP1 in two cohorts from the TCGA and GTEx databases containing 77 human ESCC samples and 1445 normal samples. Consistently, ESCC showed significantly higher expression of SPP1 (Fig. 2E). These results suggested that the expression of SPP1 protein was higher in ESCC tissues than that in adjacent normal tissues, which could elicit the production of autoantibody to SPP1 protein in ESCC.

**Table 2:** Correlation between SPP1 expression and clinicopathological features in ESCC patients

Variables	SPP1 expression in ESCC		*P*
	Low (*n* = 44)	High (*n* = 64)	
Sex			
Male	34	48	0.786
Female	10	16	
Age (years)			
< = 60	15	15	0.244
> 60	29	48	
Unknown		1	
Tumor size			
T1-T2	7	13	0.531
T3-T4	35	47	
Unknown	2	4	
Lymphatic metastasis			
Positive	26	32	0.287
Negative	17	32	
Unknown	1		
Clinical stage			
I-II	19	30	0.869
III-IV	23	34	
Unknown	2		

*ESCC* Eesophageal squamous cell carcinoma

### The positive frequency of autoantibody against SPP1 was higher in ESCC than that in normal controls

**Fig. 3:**
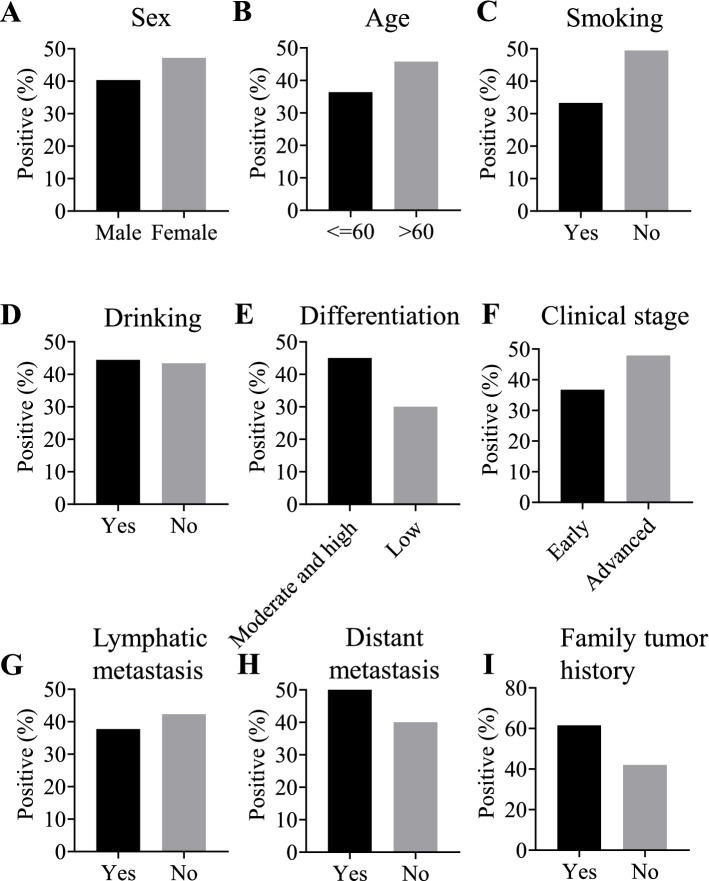
The positive frequency of anti-SPP1 autoantibody in different clinical subgroups of ESCC patients. The clinical subgroups included sex (**A**), age (**B**), smoking (**C**), drinking (**D**), differentiation (**E**), clinical stage (**F**), lymphatic metastasis (**G**), distant metastasis (**H**), family tumor history (**I**). ESCC, esophageal squamous cell carcinoma

The recombinant SPP1 protein was used as the coating antigen in ELISA to detect anti-SPP1 autoantibody in sera from ESCC patients and normal controls. The positive frequency of autoantibody to SPP1 was 45.16% (28/62) in sera from ESCC patients while it was only 16.13% (10/62) in normal human sera in discovery group, which had significantly statistical differences. The positive frequency of anti-SPP1 autoantibody was further confirmed in validation group, which also showed high positive frequency (Table 3). We further evaluate whether the positive frequency of autoantibody to SPP1 had significant differences in different clinical subgroups from ESCC (age, sex, smoking, drinking, lymphatic metastasis, TNM stage, distance metastasis, differentiation, family tumor history). The positive frequencies of autoantibody to SPP1 protein in each clinical subgroup demonstrated no differences (Fig. 3). The cut-off value was set as mean plus SD to determine a positive reaction.

**Table 3:** Frequency of autoantibody against SPP1 in human serum by ELISA

	Serum	N (%)	*P*
Discovery group	ESCC(*n* = 62)	28(45.16%)	< 0.001
	NC(*n* = 62)	10(16.13%)	
Validation group	ESCC(*n* = 100)	41(41.00%)	< 0.001
	NC(*n* = 100)	13(13.00%)	

*ESCC* Esophageal squamous cell carcinoma, *NC* Normal controls

### Autoantibody against SPP1 was relatively increased in ESCC compared to normal controls

**Fig. 4:**
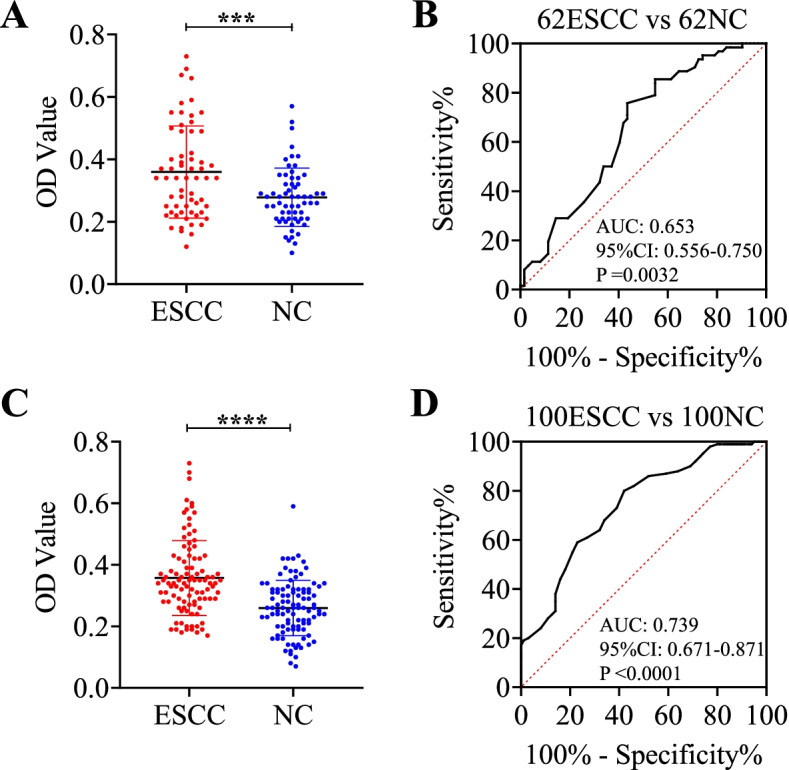
Serum anti-SPP1 autoantibodies of ESCC patients and normal controls in discovery group and validation group. **A**, **C** The distribution of sera anti-SPP1 autoantibodies in ESCC and NC in discovery group (**A**) and validation group (**C**). **B**, **D** ROC curve analysis of serum anti-SPP1 autoantibodies for distinguishing ESCC from NC in discovery group (**B**) and validation group (**D**). ESCC, esophageal squamous cell carcinoma; NC, normal controls; ROC, receiver operating characteristic. ***P < 0.01; ****P < 0.001

Firstly, the levels of autoantibody to SPP1 in sera were detected by ELISA in discovery group with ESCC patients (*n* = 62) and normal controls (*n* = 62). Compared to normal controls, autoantibody to SPP1 was significantly increased in patients with ESCC (Fig. 4A). Subsequently, ROC was generated to evaluate the potential significance of autoantibody to SPP1 as a novel biomarker for ESCC detection. ROC analysis demonstrated that autoantibody to SPP1 was obvious to distinguish patients with ESCC from NC, which had an AUC of 0.653 (95%CI: 0.556–0.750) with the sensitivity (Se) of 45.16% and specificity (Sp) of 83.87% (Fig. 4B, Table 4).

**Table 4:** Autoantibody against SPP1 in the diagnosis of ESCC

Cohorts	AUC	95%CI	Se (%)	Sp (%)	YI	PLR	NLR	PPV (%)	NPV (%)	Accuracy (%)
Discovery group	0.653	0.556–0.750	45.16	83.87	0.29	2.8	0.65	73.68	60.47	64.52
Validation group	0.739	0.671–0.807	41.00	87.00	0.28	3.15	0.68	75.93	59.59	64.00

*AUC* Area under the curve, *CI* Confidence interval, *Se* Sensitivity, *Sp* Specificity, *YI* Youden index, *PLR* Positive likelihood ratio, *NLR* Negative likelihood ratio, *PPV* Positive predictive value, *NPV* Negative predictive value

To further confirm the reproducibility of serum anti-SPP1 autoantibody as a novel biomarker in ESCC patients’ detection. The levels of serum autoantibody to SPP1 were detected in a validation group with 100 ESCC patients and 100 normal controls. Compared with normal controls, we found a significantly higher level of autoantibody response to SPP1 in patients with ESCC (Fig. 4C). Consistently, autoantibody to SPP1 in validation group could obviously discriminate patients with ESCC from NC since the AUC was 0.739 (95%CI: 0.671–0.871) with the sensitivity of 41.00% and the specificity of 87.00% (Fig. 4D, Table 4). Besides, Youden index (YI), predictive value (PV), likelihood ratio (LR) and accuracy were shown in Table 4.

### Western blotting confirmed the ELISA results

**Fig. 5:**
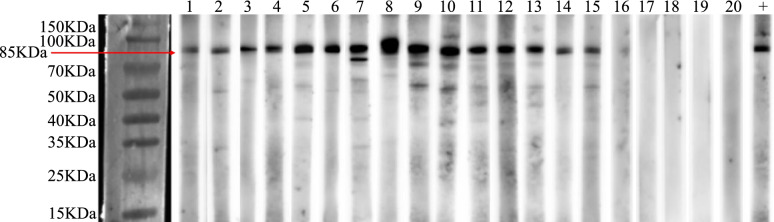
Western blotting of anti-SPP1 autoantibody in sera from 15 ESCC patients and 5 normal controls. Lanes 1–15, the cropping strips of 15 representative ESCC sera had strong reactivity with SPP1 recombinant protein, which were also positive in ELISA. Lanes 16–20, the cropping strips of 5 random normal human sera with negative reactivity to SPP1 recombinant protein. Lane 21, anti-SPP1 antibody used as the positive control. SPP1, secreted phosphoprotein 1. ESCC, esophageal squamous cell carcinoma

To further confirm the results of ELISA, we performed western blotting with 15 ESCC sera which were positive in ELISA and 5 normal human sera randomly selected from the discovery and validation groups. The positive control was regarded as quality control. As shown in Fig. 5, the 15 representative ESCC sera showed strong response to SPP1 recombinant protein, which were also positive in ELISA, while 5 normal human sera showed negative response to SPP1 recombinant protein, which were also negative in ELISA. Full-length blots of all strips are presented in Supplementary Fig. 2. The results of western blotting were consistent with the results of ELISA and confirmed the occurrence of immunoreactivity to SPP1 in sera.

### Anti-SPP1 autoantibody was significantly higher in ESCC patients with family tumor history

**Fig. 6:**
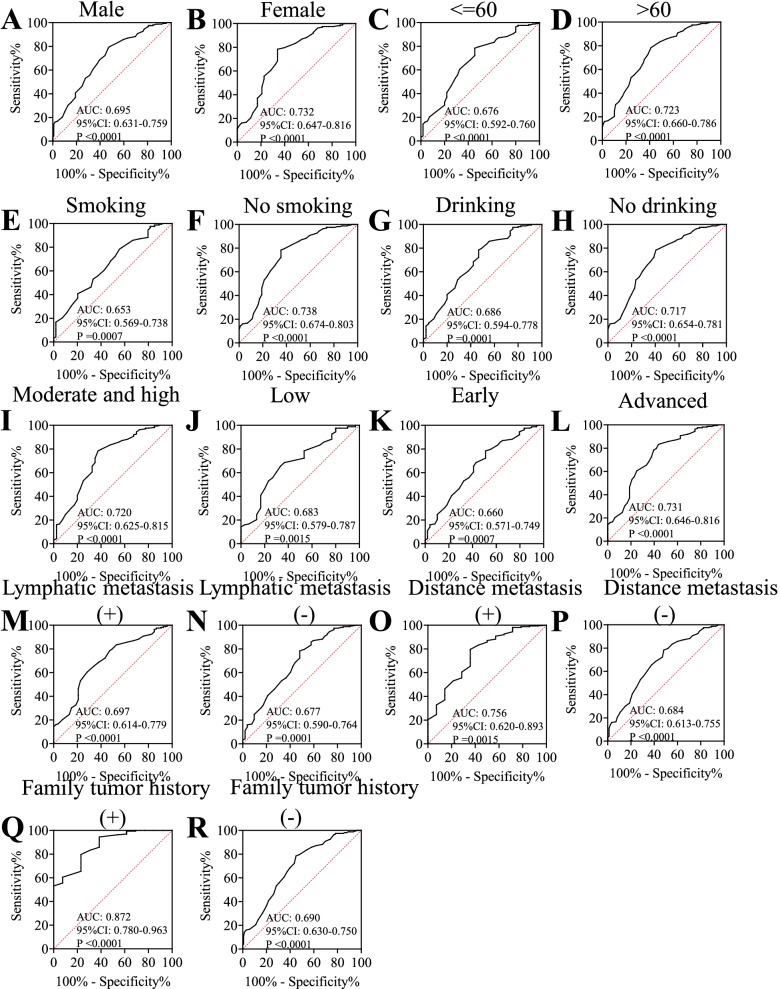
The ROC curve analysis of anti-SPP1 autoantibody in different clinical subgroups from ESCC patients. The clinical subgroups included male (**A**) and female (**B**), <  = 60 (**C**) and > 60 (**D**), smoking (**E**) and no smoking (**F**), drinking (**G**) and no drinking (**H**), Moderate and high differentiation (**I**) and low differentiation (**J**), early stage (**K**) and advanced stage (**L**), lymphatic metastasis ( +) (**M**) and lymphatic metastasis (-) (**N**), distance metastasis ( +) (**O**) and distance metastasis (-) (**P**), family tumor history ( +) (**Q**) and family tumor history (-) (**R**). ROC, receiver operating characteristic; ESCC, esophageal squamous cell carcinoma

We divided 162 ESCC patients from the discovery and validation groups into different clinical subgroups according to different variables and tried to explore the levels and the AUCs of anti-SPP1 autoantibody in different subgroups of ESCC, including age, sex, smoking, drinking, differentiation, TNM stage, lymphatic metastasis, distance metastasis and family tumor history. Firstly, Table 5 illustrated that serum level of anti-SPP1 autoantibody was significantly higher in ESCC patients with family tumor history than that in the group without family tumor history (*P* < 0.01). For other clinicopathologic characteristics, the levels of autoantibody against SPP1 showed no significant difference. Next, the AUCs of ESCC patients with or without family tumor history had statistically significant difference (*P* < 0.05) while there was no difference in other clinical subgroups in Fig. 6. These results suggested the potential significance of anti-SPP1 autoantibody as a biomarker in detection of ESCC with family tumor history.

**Table 5:** Relationship between clinicopathological factors and autoantibodies against SPP1 in ESCC patients

		Anti-SPP1	*P*
		Mean ± SD (OD values)	
Mean age ± SD	63.5 ± 8.2		
Age range	42–88		
< = 60	55	0.3320 ± 0.1079	0.051
> 60	107	0.3712 ± 0.1412	
Sex			
Male	109	0.3491 ± 0.1242	0.223
Female	53	0.3760 ± 0.1461	
Smoking			
Yes	54	0.3402 ± 0.1362	0.165
None	99	0.3718 ± 0.1330	
Drinking			
Yes	45	0.3587 ± 0.1454	0.903
None	106	0.3616 ± 0.1310	
Differentiation			
Moderate and high	40	0.3728 ± 0.1520	0.344
Low	30	0.3407 ± 0.1207	
TNM stage			
I-II	49	0.3378 ± 0.1330	0.286
III—IV	48	0.3665 ± 0.1303	
Lymphatic metastasis			
Positive	53	0.3404 ± 0.1142	0.600
Negative	52	0.3537 ± 0.1430	
Distance metastasis			
Positive	14	0.3814 ± 0.1309	0.346
Negative	85	0.3458 ± 0.1305	
Family tumor history			
Yes	13	0.4623 ± 0.1587	0.004
None	138	0.3497 ± 0.1283

## Discussion

ESCC still has poor prognosis mainly due to lacking of effective diagnostic biomarkers. In this study, we found that the expression of SPP1 protein was significantly higher in ESCC tissues than that in adjacent normal tissues. The levels of serum autoantibody against SPP1 were significantly higher in patients with ESCC compared to NC in both discovery (62 ESCC VS 62 NC) and validation groups (100 ESCC VS 100 NC). Autoantibody to SPP1 was obvious to distinguish patients with ESCC from NC with the AUCs of 0.653 and 0.739 in discovery and validation group respectively, suggesting that serum anti-SPP1 autoantibody had potential significance to be a novel biomarker for ESCC detection.

SPP1 plays an important role in cancer progression [27–29]. The upregulation of SPP1 enhances PDL1 expression and facilitates immune invasion of lung cancer [28]. SPP1 promotes the migration, invasion and cisplatin resistance of lung cancer cells, and overexpression of SPP1 is correlated with tumor grade and poor clinical prognosis [30]. In addition, SPP1 could promote proliferation and inhibit apoptosis in head and neck squamous cell carcinoma [30]. A study indicated that SPP1 is closely related with evolution of tumor cell and change of microenvironment in hepatocellular carcinoma, suggesting that SPP1 may be a key regulator in treatment of cancer [31]. SPP1-CD44 axis could promote cancer stemness in pancreatic cancer [32]. It was reported that lacking of SPP1 inhibits progression by mediating the PI3K/Akt signaling pathway in tongue cancer [33]. Besides, high expression of SPP1 is correlated with poor survival in several cancers [34, 35]. Here, we found that SPP1 protein was highly expressed in ESCC tissues than that in adjacent normal tissues by IHC analysis. Based on the above evidence and the results of IHC, we found that SPP1 is involved in the occurrence of tumor, and thus it could be a tumor-associated antigen occurring in ESCC.

TAAs can be secreted into the blood of patients, which induce immune responses and produce autoantibodies against the TAAs [36]. Anti-TAAs autoantibodies can be detected before the occurrence of clinical symptoms and have great potential to be serum biomarkers for the detection of cancers [37, 38]. There are several autoantibodies reported as serum biomarkers for ESCC patients’ detection. It was reported that anti-Fascin autoantibody was detected in sera from 149 ESCC and 98 NC with the AUC of 0.636. However, this study lacked further validation in another independent group and did not confirm the results of ELISA by western blotting [39]. It was shown that serum anti-MMP7 autoantibody could detect ESCC with the AUC of 0.87, sensitivity of 78% and the specificity of 81% in sera from 50 patients with ESCC and 58 NC, whereas the serum samples in this study were not enough and also lacked further validation [40]. Compared with other studies on evaluating serum autoantibody as a novel biomarker in detection of patients with ESCC, our study had some advantages. Firstly, anti-SPP1 autoantibody could distinguish ESCC patients from normal controls in discovery and validation groups, which made the results of ELISA more dependable. Secondly, western blotting further confirmed the results of ELISA. Thirdly, the elevated anti-SPP1 autoantibody in ESCC sera was consistent with the overexpression of SPP1 protein in ESCC tissue, which made a speculation that the overexpression may trigger strong immune response of SPP1 autoantibody in ESCC patients. Therefore, we demonstrated that autoantibody to SPP1 is a potential biomarker in detection of patients with ESCC.

Upon the availability of clinicopathological features in 162 ESCC patients from the discovery and validation groups, we explore the levels and the AUCs of anti-SPP1 autoantibody in different subgroups of ESCC patients. Then we found the interesting information that serum level of anti-SPP1 autoantibody was significantly higher in ESCC patients with family tumor history, which could distinguish ESCC patients with family tumor history from that without family tumor history. This is partially consistent with similar finding that the inheritance of defective BRCA1 or BRCA2 allele predisposes an individual to develop breast cancer [41]. Our studies indicated that increased anti-SPP1 autoantibody may be more likely to develop ESCC for people with family tumor history. However, more studies are needed to further confirm the findings.

In summary, it is the first study to detect anti-SPP1 autoantibody in ESCC. Our findings provide the evidence that anti-SPP1 autoantibody was significantly elevated in patients with ESCC, which was identify with the overexpression of its matching antigen in ESCC tissues. The diagnostic values of autoantibody to SPP1 in ESCC were verified by two groups to present reliable results, and the results from western blotting were in line with the results of ELISA. These suggested that autoantibody to SPP1 had potential significance to be a novel serum biomarker for detection of patients with ESCC.

## Supplementary Information

**Additional file 1: Table S1.** The clinicopathologic characteristics in 108 ESCC patients.

**Additional file 2.** Microscopy images of Immunohistochemistry from ESCC tissue microarray.

**Additional file 3.** The uncropped images of western blotting strips of 20 sera in ELISA and a positive control.

## Data Availability

The datasets used and/or analyzed during the current study are available from the corresponding author on reasonable request.

## Abbreviations

SPP1: Secreted phosphoprotein 1
ESCC: Esophageal squamous cell carcinoma
EAC: Esophageal adenocarcinoma
EC: Esophageal cancer
NC: Normal controls
IHC: Immunohistochemistry
ELISA: Enzyme-linked immunosorbent assay
AUC: Area under curve
TAAs: Tumor-associated antigens
PD-L1: Programmed death ligand 1
HRP: Horseradish peroxidase
OD: Optical density
ROC: Receiver operating characteristic
SD: Standard deviation
CI: Confidence interval
Se: Sensitivity
Sp: Specificity
YI: Youden index
PLR: Positive likelihood ratio
NLR: Negative likelihood ratio
PPV: Positive predictive value
NPV: Negative predictive value
