# Supplementary material for: Serum anti-SPP1 autoantibody as a potential novel biomarker in detection of esophageal squamous cell carcinoma
Source: BMC Cancer. 2022 Aug 29;22:932. doi: 10.1186/s12885-022-10012-9 (PMC9425987; doi:10.1186/s12885-022-10012-9)
Supplement: Supplementary file 1 — Additional file 1: Table S1. The clinicopathologic characteristics in 108 ESCC patients. [file 12885_2022_10012_MOESM1_ESM.pdf]

Table S1. The clinicopathologic characteristics in 108 ESCC patients.

Variables	N(%)
Age (years)	
<=60	30(27.78)
>60	77(71.29)
Unknow	1(0.93)
Sex	
Male	82(75.93)
Female	26(24.07)
Pathological grades	
G1	40(37.04)
G2	49（45.37）
G3	19（17.59）
Lymphatic metastasis	
Positive	58（53.70）
Negative	49（45.37）
Unknow	1（0.93）
Distant metastasis	
Positive	0（0.00）
Negative	108（100）
AJCC stage	
I	4（3.70）
II	45（41.67）
III	54（50.00）
IV	0（0.00）
Unknow	5（4.63）

AJCC, American Joint Committee on Cancer; ESCC, esophageal squamous cell carcinoma.
