# Supplementary material for: Serum anti-SPP1 autoantibody as a potential novel biomarker in detection of esophageal squamous cell carcinoma
Source: BMC Cancer. 2022 Aug 29;22:932. doi: 10.1186/s12885-022-10012-9 (PMC9425987; doi:10.1186/s12885-022-10012-9)

Microscopy images of Immunohistochemistry from ESCC tissue microarray.

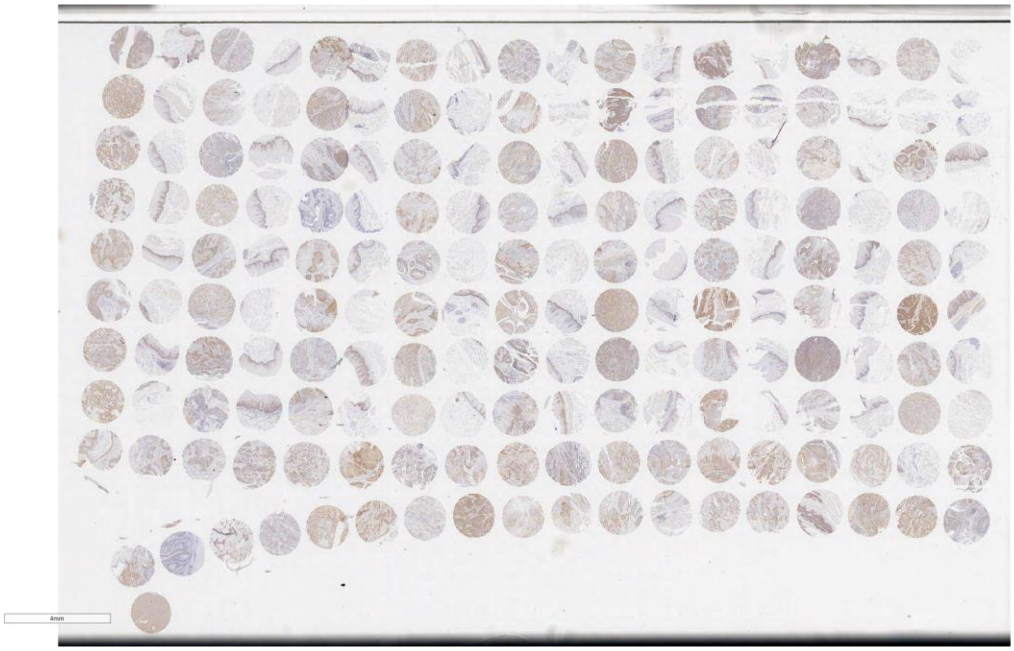

Figure 2A Representative IHC staining images of SPP1 in adjacent normal tissue and tumor tissue from ESCC tissue microarray.

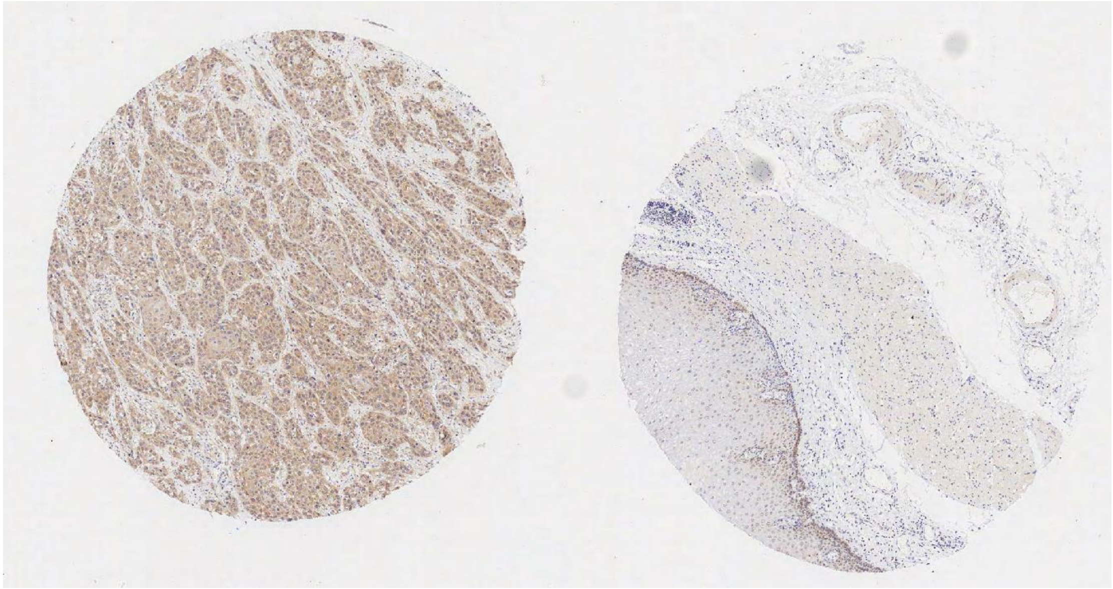

Supplement: Supplementary file 2 — Additional file 2. Microscopy images of Immunohistochemistry from ESCC tissue microarray. [file 12885_2022_10012_MOESM2_ESM.pdf]
