# Supplementary material for: Serum anti-SPP1 autoantibody as a potential novel biomarker in detection of esophageal squamous cell carcinoma
Source: BMC Cancer. 2022 Aug 29;22:932. doi: 10.1186/s12885-022-10012-9 (PMC9425987; doi:10.1186/s12885-022-10012-9)
Supplement: Supplementary file 3 — Additional file 3. The uncropped images of western blotting strips of 20 sera in ELISA and a positive control. [file 12885_2022_10012_MOESM3_ESM.pdf]

The uncropped images of western blotting strips of 20 sera in ELISA and a positive control.

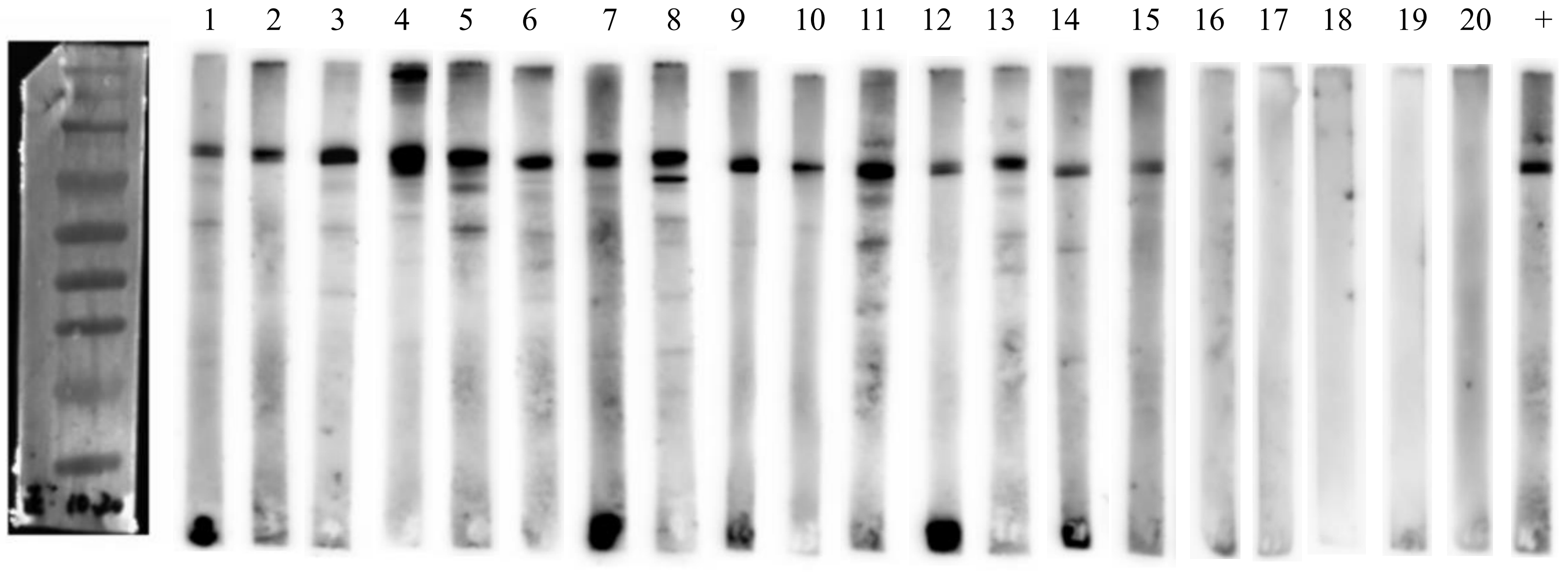

In the experiment of western blotting, the recombinant SPP1 protein was electrophoresed by 10% SDS-PAGE and transferred onto a nitrocellulose membrane that was then cut into strips and incubated with selected sera diluted at 1:100 respectively, subsequently incubated with mouse anti-human IgG conjugated HRP diluted at 1:5000. Finally, the reaction signal of each strip was obtained by azure biosystems.
